# Tin and Tin Compound Materials as Anodes in Lithium-Ion and Sodium-Ion Batteries: A Review

**DOI:** 10.3389/fchem.2020.00141

**Published:** 2020-03-19

**Authors:** Haoyi Mou, Wei Xiao, Chang Miao, Rui Li, Liming Yu

**Affiliations:** College of Chemistry and Environmental Engineering, Yangtze University, Jingzhou, China

**Keywords:** tin, tin compound, anode, lithium-ion batteries, sodium-ion batteries

## Abstract

Tin and tin compounds are perceived as promising next-generation lithium (sodium)-ion batteries anodes because of their high theoretical capacity, low cost and proper working potentials. However, their practical applications are severely hampered by huge volume changes during Li^+^ (Na^+^) insertion and extraction processes, which could lead to a vast irreversible capacity loss and short cycle life. The significance of morphology design and synergic effects-through combining compatible compounds and/or metals together-on electrochemical properties are analyzed to circumvent these problems. In this review, recent progress and understanding of tin and tin compounds used in lithium (sodium)-ion batteries have been summarized and related approaches to optimize electrochemical performance are also pointed out. Superiorities and intrinsic flaws of the above-mentioned materials that can affect electrochemical performance are discussed, aiming to provide a comprehensive understanding of tin and tin compounds in lithium(sodium)-ion batteries.

## Introduction

Since the commercialization of lithium-ion batteries (LIBs) by the Sony Corporation in 1991, LIBs are widely used in portable devices, electric vehicles and energy storage equipment for their benefits of having no memory effect, long cycle life and high energy density (Tarascon and Armand, 2010; Kim et al., 2012; Wang et al., 2019). With largely depleting lithium resources, the existing limited and unevenly distributed lithium reserves cannot meet the increasing demands of LIBs (there is an estimated 17 ppm in the earth's crust; Grosjean et al., 2012). Due to abundant sodium reserves (there is an estimated 23,000 ppm in the earth's crust), sodium-based batteries can be an attractive alternative. Traditional Na-S batteries require operating temperatures between 300 and 350°C to allow sufficient Na^+^ conductivity of NaAl_11_O_17_, but safety issues and energy loss from maintaining the operating temperature are inevitable (Wen et al., 2008; Xin et al., 2014; Kou et al., 2019). Motived by the similar chemical properties of sodium and lithium, researchers have shifted their attention to ambient temperature sodium-ion batteries (SIBs), but lots of problems need to be addressed for the practical application of SIBs (Yabuuchi et al., 2014; Li et al., 2018; Wu L. et al., 2018; Liu Y. et al., 2019). The main issue is the larger radius size of Na^+^ (1.09 Å) compared with Li^+^ (0.74 Å), which brings about sluggish reaction kinetics with low capacity, poor rate capability, and short cycling life (Chevrier and Ceder, 2011; Xu et al., 2013; Li et al., 2018). Extensive studies have been carried out to understand the requirements of commercial SIBs, which are great choices for low cost and large-scale energy storage equipment required for intermittent renewable energy and smart grids (Palomares et al., 2012; Pan et al., 2013). Comparitively, the energy density of LIBs cannot fully satisfy an increasingly growing need for electronic energy storage devices (Xiao et al., 2018; Fang et al., 2020). The present conventional anode in LIBs is graphite, which follows a intercalation/de-intercalation reaction pathway with a low theoretical capacity (378 mAh/g) and is electrochemically unfavorable for SIBs owing to the larger size of Na^+^ (Qian et al., 2014). Therefore, not all successful experiences from LIBs are viable to be applied in SIBs. Usually, graphene and non-graphitic carbon (like hard carbon and carbon black) are conventional anodes in SIBs. Additionally, TiO_2_, Na_2_Ti_3_O_7_, Sn, SnO_2_, SnS_2_, Sb, and P, etc. are potential anode materials for Na^+^ storage in SIB systems (Slater et al., 2013; Li et al., 2018; Guan et al., 2020). Thanks to a similar charging-discharging mechanism, tin-based anodes' alloying/dealloying reactions have attracted considerable attention because they are applicable to both LIBs and SIBs with a high theoretical capacity (Stevens and Dahn, 2000; Zhu et al., 2013). Environmental benignity, low costs, and lower operating potentials than graphite are also attractive features for tin and tin compounds, but they contain the following intrinsic defects (Fu et al., 2016). Tin and tin compounds as anodes in LIBs (SIBs) sustain colossal volume changes during Li^+^ (Na^+^) insertion and extraction processes, which leads to pulverization of the active materials as well as losing electrical contact with the collector (Zhang, 2011; Liu D. et al., 2019). Moreover, a continuously regenerated solid electrolyte interphase (SEI) layer between the electrode and electrolyte interface will consume extra lithium (sodium) ions, causing large irreversible capacity loss and poor cycle stability (Beaulieu et al., 2001). Last but not least, the electronic conductivity of SnO_2_ (0.1 S/m) and SnS_2_ (1 S/m) is much inferior to Sn (9.1 × 10^6^ S/m) (Thangaraju and Kaliannan, 2000; Saadeddin et al., 2006; Nie et al., 2020). To cope with these problems, many measures have been taken and summarized as follows.

Firstly, according to comprehensive investigations nano-scale tin and tin compounds can alleviate the inter stress brought on by volume changes, to some extent, and can shorten the transfer paths of lithium (sodium) ions and electrons. Additionally, more reactive sites on the interface between electrodes and electrolytes are generated (Uchiyama et al., 2008; Park and Park, 2015; Park et al., 2018). The second method is to incorporate tin and tin compounds with one or more stress-accommodating phases that have can assure electronic conductivity, such as carbonaceous materials, metals and some transitional metal compounds (Kepler et al., 1999; Takamura et al., 1999). In 2005, Sony commercialized the first tin-based amorphous anode with the trademark “Nexelion” and this anode is composed of Sn, Co and C, where Co and C are identified as conductive and stress-releasing phases. According to Sony, Nexelion has a capacity of 900 mAh, which is 28 % higher than conventional graphite (700 mAh) at 0.2°C. Extensive investigations have been made to find a feasible and low-cost way to synthesize tin- and tin compound-based anodes with satisfactory physicochemical and electrochemical properties for both LIBs and SIBs at the same time. In this review, we focus on the recent progress of Sn, SnO_2_, and SnS_2_ as anodes in LIBs and SIBs. This comprehensive review provides an in-depth account of the similarities and differences between Sn, SnO_2_, and SnS_2_ as used in LIBs (SIBs) as well as clear directions for the structure design and fabrication procedures regarding anode material syntheses in LIBs and SIBs.

## Tin and Tin Compounds in LIBs

### Sn-Based Composites

Sn has a high theoretical specific capacity of 993.4 mAh/g, according to the reversible reaction Sn+*x*Li^+^+*x*e^−^↔Li_*x*_Sn (0≤*x*≤4.4) (Lee et al., 2003). However, huge volume changes and aggregation of Sn particles during the alloying/dealloying process are the main obstacles for practical applications (Beaulieu et al., 2001). Generally, carbonaceous materials and Sn-based intermetallics are believed to address these issues efficiently and largely improve the battery performance of Sn-based anode materials (Zhao et al., 2015; Ying and Han, 2017). Carbon materials, either acting as the support or coating, can effectively ease volume changes and aggregation of Sn particles and increase the overall conductivity, especially with graphene (Wen et al., 2016). Zhou et al. have reported a high-performance anode where tin nanoparticles are impregnated into nitrogen-doped graphene (Zhou et al., 2013a). The graphene coating can facilitate electron transport and prevent aggregation of tin particles. Add void spaces between graphene and tin nanoparticles avail the accommodation of volume changes. As a result, the final composite delivers a reversible capacity of 481 mAh/g at a current density of 100 mA/g.

Some Sn-based intermetallics have also been considered as a promising choice, such as Sn-Cu, Sn-Co, Sn-Sb, Sn-Bi, Sn-Se, Sn-Fe and Sn-Ni etc (Yang et al., 1999; Yoon et al., 2009; Xue et al., 2010; Dang et al., 2015; Qin et al., 2017). Among all these types of intermetallics, Sony's Nexelion-consisting of Sn, Co, and C-is the first commercialized tin-based anode, but the composition is not fully revealed. Hence, it is important to further investigate the role and mechanism of cobalt in the Sn-Co intermetallic system. In principle, cobalt is considered an inactive component used to buffer the volume changes. However, according to the systematic study of Sn_1−*x*_Co_*x*_ (0<*x*<0.6) and [Sn_0.55_Co_0.45_]_1−*y*_C_*y*_ (0<*y*<0.5) conducted by Dahn et al., the Sn_1−*x*_Co_*x*_ system is amorphous when 0.28 <*x*<0.43 and an amorphous structure can hold part of the capacity in place of alloying anodes in LIBs. In addition, cobalt does not form intermetallic Co-carbides which avoids the exclusion of crystalline tin, improving the cycle stability of the composite (Tamura et al., 2004; Dahn et al., 2006; Todd et al., 2007; Li et al., 2011).

Sn-Cu alloy is another extensively explored anode in LIBs, especially in the stable Cu_6_Sn_5_ intermetallic phase. According to the detailed *in-situ* X-ray study of Cu_6_Sn_5_ by Larcher and his coworker, the two reverse phase transitions of Cu_6_Sn_5_ reacting with Li^+^ are listed as follows (Larcher et al., 2000):

(1) $$\begin{array}{l} \text{C}{\text{u}}_{6}\text{S}{\text{n}}_{5}↔\text{L}{\text{i}}_{2}\text{CuSn} \end{array}$$

(2) $$\begin{array}{l} \text{L}{\text{i}}_{2}\text{CuSn}↔\text{L}{\text{i}}_{4.4}\text{Sn}+\text{Cu} \end{array}$$

As the Cu content in the Cu-Sn alloy increases, the final obtained product will significantly improve in cyclability, because Cu is used as an inactive buffering matrix to relieve the volume expansion. However it also results in a relatively lower discharge capacity, for example, the theoretical discharge specific capacity of Cu_6_Sn_5_ in LIBs is 584 mAh/g (Trahey et al., 2009). Core/shell Cu_6_Sn_5_@SnO_2_-C anode materials are prepared by boiling Sn and Cu powders in a sucrose solution with air, as reported by Hu's group, in which Cu_6_Sn_5_ as an inert foundation replaces the electrochemically inactive Cu, SiC and Ni (Hu et al., 2015). As a consequence, the composite exhibits a high discharge specific capacity of 619 mAh/g at 1.0°C after 500 cycles, and SEM images before and after the first cycle show that the maximum volume change ratio decreases to 12.7%.

On the other hand, some Sn-based intermetallics with electrochemically active metals, like Sb, Bi, and Ge, have shown higher initial capacities and better electrochemical properties than the individual active materials, which is due to the different potentials *vs*. Li^+^/Li of these active metals. The temporarily separated charge-discharge process of these active materials guarantees that Sn and the electrochemically active metals can operate as volume-releasing phases for each other alternately (Trifonova et al., 2002; Zhang, 2011). He and his co-workers have reported a colloidal synthesis of monodisperse SnSb nanocrystals that deliver high specific capacities of 700 and 600 mAh/g at 0.5 and 4.0°C after 100 cycles, respectively (He et al., 2015).

**Figure 1:**
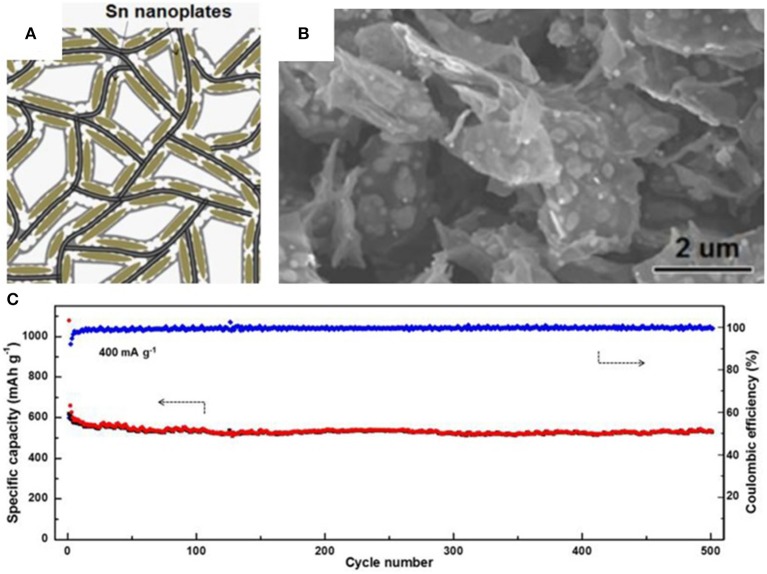
Schematic illustration **(A)** and SEM image **(B)** of tin nanoplates encapsulated in foam like graphene backboned carbonaceous carbon matrix (F-G/Sn@C), cycling performance **(C)** of F-G/Sn@C at 400 mA/g from 0.01 to 2.00 V. Reproduced from Luo B. et al. (2016) with permission from Copyright (2016) Elsevier.

Graphene with its excellent electrical conductivity, flexibility, and high specific surface area can be an ideal buffering matrix for tin-based anodes (Li and Kaner, 2008). In 2015, Luo et al. synthesized a novel anode where tin nanoparticles were encapsulated into graphene backboned carbon foam (Luo B. et al., 2016). Graphene and the outermost carbon coating serve as a physical boundary to prevent the aggregation of well-distributed tin nanoparticles and alleviate the huge volume changes of tin particles. The unique structure is prepared by uniformly growing SnO_2_ on the surface of graphene oxide and coating with porous carbon through a hydrothermal processes, finally calcinating in a reducing atmosphere. The resulting composite shows excellent cycle stability and exceptional rate performance in LIBs as well as in SIBs. A reversible specific capacity of 506 mAh g^−1^ can be achieved at a current density of 400 mAh/g and retained at 270 mAh/g, and even at 3,200 mA/g after 500 cycles (Figure 1). A summary of anode materials, synthetic methods, and electrochemical performance in tin-based anode composites is shown in Table 1 for comparison.

**Table 1:** Anode materials, synthetic methods and electrochemical performance of a Sn-based anode.

**Anode materials**	**Synthetic method**	**ICE (%)**	**Cyclability (mAh/g)**	**Rate performance (mAh/g)**	**References**
Graphene/Sn@carbonaceous foam	Hydrothermal method and thermal reduction	About 60	777 (100 cycles at 100 mA/g)	506 (500 cycles at 400 mA/g) 270 (500 cycles at 3200 mA/g)	Luo B. et al., 2016
Sn@N-doped carbon	*In situ* polymerization and carbon thermal reduction	78.5	788 (300 cycles at 100 mA/g)	522 (1,000 cycles at 500 mA/g)	Chang et al., 2017
CoSn_2_/*a*-TiC/C	Ball milling	83.5	479 (180 cycles at 100 mA/g)	380 (500 mA/g)	Park et al., 2018
Core/shell Cu_6_Sn_5_@SnO_2_-C	Ball milling and heat treatment	65	619 (500 cycles at 200 mA/g)	390 (2 A/g)	Hu et al., 2015
Sn@hollow carbon cube	Combination of *in situ* chemical synthesis in aqueous solution, chemical vapor deposition (CVD) and acid etching	About 55	624 (200 cycles at 600 mA/g)	537 (1,000 cycles at 3 A/g)	Huang et al., 2015
C/Sn/C hollow spheres	*In situ* chemical synthesis in organic solution	62	1,100 (130 cycles at 100 mA/g)	430 (at 5 A/g)	Sun et al., 2019
Si@Sn-MoF	*In situ* chemical synthesis in organic solution	60.6	1,360 (250 cycles at 200 mA/g)	618 (800 cycles at 2 A/g)	Zhou et al., 2019
Sn@3D graphene networks	Freeze drying and chemical vapor deposition (CVD)	69	1,089 (100 at 200 mA/g)	459 (at 5 A/g) 270 (at 10 A/g)	Qin et al., 2014
Ni_3_Sn_2_ microcages	Solvothermal reduction and crystallization	58.9	696 (400 cycles at 0.2 C) 530 (1,000 cycles at 1 C)	404 (at 10 C) 404 (at 10 C)	Liu J. et al., 2014
SnSb@N-doped carbon fiber	Electrospinning	72.2	892.6 (100 cycles at 100 mA/g)	487 (at 2 A/g)	Yuan et al., 2018

*ICE, Initial coulombic efficiency*.

### SnO_2_-Based Composites

Tin oxide materials were first discovered and applied in LIBs with a high specific capacity by Idato et al. from Fuji Photo Film in 1997 (Idota et al., 1997). From then on, SnO_2_-based anodes in LIBs have drawn considerable attention because of their high theoretical capacity, resource availability, environmental benignity, and low operating potentials (0.3 and 0.5 V vs. Li^+^/Li in charge and discharge processes; Li R. et al., 2019). The chemical reactions of SnO_2_ with lithium electrodes involve the following two steps (Courtney and Dahn, 1997; Chen and Lou, 2013; Zhou et al., 2013b):

(3) $$\begin{array}{l} \text{Sn}{\text{O}}_{2}+4\text{L}{\text{i}}^{+}+4{\text{e}}^{-}\to \text{Sn}+2\text{L}{\text{i}}_{2}\text{O} \end{array}$$

(4) $$\begin{array}{l} \text{Sn}+\text{xL}{\text{i}}^{+}+\text{x}{\text{e}}^{-}\to \text{L}{\text{i}}_{\text{x}}\text{Sn}\left(0\leq \text{x}\leq 4.4\right) \end{array}$$

The theoretical specific capacity for bulk SnO_2_ electrodes is 780 mAh/g, which includes conversion reactions and further alloying/dealloying reactions. It is worth noting that the conversion reactions of bulk SnO_2_ to Sn are irreversible but can be partly reversible for nanosized SnO_2_ and the theoretical specific capacity can be up to 1,484 mAh/g (Kim et al., 2005; Zhang et al., 2009). Like Sn, the as-formed Sn from SnO_2_ suffers from huge volume changes (250%) in alloying/dealloying processes and what's worse, the inner stress originating from volume changes causes pulverization of the SnO_2_ electrodes. The conversion reaction and pulverization of the SnO_2_ electrode brings about a severe capacity decrease in the SnO_2_. Another issue that needs to be mentioned is that the Sn particles from conversion reactions tend to agglomerate into Sn clusters that will weaken the electrochemical activity (Park et al., 2007; Deng et al., 2016). These flaws are the main limitations for the commercialization of SnO_2_-based anodes in LIBs.

To deal with the defects of SnO_2_-based electrodes, the adopted strategies are summarized as follows. The first strategy is to convert bulk SnO_2_ particles into nanosized particles and simultaneously design nanostructures such as nanospheres, nanotubes, and nanofilms (Liu et al., 2016). The nanostructures can accommodate volume changes and shorten the diffusion length for electrons and lithium ions, but the accompanying negative effect for nanostructure materials is that the high surface energy will lead to the agglomeration of nanoparticles, which is electrochemically unfavorable (Chen and Lou, 2013). Additionally, structure design alone cannot compensate for the whole volume change whilst producing the desired electrochemical performance. Hence, another strategy is proposed, which is to combine the designed architecture with carbonaceous materials including carbon nanotubes, amorphous carbon, hard carbon, and graphene (Read et al., 2001; Yang et al., 2013; Zhou et al., 2016). Carbonaceous materials not only prevent nano SnO_2_ and as-formed Sn grains from agglomeration by creating a physical barrier, but they also improve the overall electronic conductivity of the SnO_2_-based composite.

When it comes to size control of SnO_2_ in LIBs, it is not found that as the SnO_2_ particles get smaller, the better the electrochemical performance becomes. As the size of SnO_2_ particles decreases, the SEI layer becomes larger, which hinders SnO_2_ from reacting with lithium ions (Kim et al., 2013). According to Ahn et al., the optimum size of colloidal synthesis of SnO_2_ particles is ~11 nm during Li^+^ insertion/extraction processes (Ahn et al., 2004). A series of sizes of SnO_2_ hollow spheres as investigated by Kim et al. demonstrated that SnO_2_ hollow spheres with a size of 25 nm showed the best electrochemical performance (750 mAh/g after 50 cycles at a current density of 100 mA/g; Kim et al., 2013). Moreover, SnO_2_ nanoparticles synthesized via the hydrothermal method with a size of 3 nm deliver the best reversible capacity (740 mAh/g after 60 cycles at 1,800 mA/g) compared to the ones at 4 and 8 nm (Kim et al., 2005). As a consequence, the optimum size for SnO_2_ nanoparticles varies for different fabrication processes.

**Figure 2:**
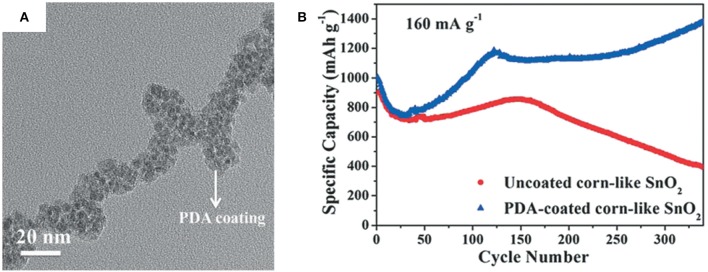
TEM image **(A)** of PDA-coated SnO_2_ and cycling performance **(B)** of PDA-coated corn-like SnO_2_ and uncoated corn-like SnO_2_ at 160 mA/g. Reproduced from Jiang B. et al. (2017) with permission from Copyright (2017) WILEY-VCH.

Recently, Jiang et al. have shown that well-designed cob-like SnO_2_ nanoparticles coated with polydopamine and prepared by a hydrothermal processes exhibit an excellent rate capability and a long cycle life at around 1,400 mAh/g at a current density of 160 mA/g after 300 cycles (Jiang B. et al., 2017). Bush-like hydroxypropyl cellulose-graft-poly(acrylic acid) (HPC-g-PAA) and Na_2_SnO_3_·3H_2_O were used as the template and SnO_2_ precursor, respectively. SnO_2_ particles with an average size of 5 nm were uniformly grown on the graft of HPC-g-PAA template, and gaps of 3–5 nm among SnO_2_ particles could be observed, which allowed it to accommodate for volume changes of SnO_2_ particles in the electrode. Moreover, the final carbonized polydopamine coating was shown to help form stable SEI layers, which is helpful to enhance the cycle stability (Figure 2).

Beyond the use of carbon, transition metal compounds are also regarded as an effective component to be introduced into SnO_2_ electrodes with syngeneic effects of combined materials. TiO_2_, for example, is a very stable LIB anode material because of its outstanding electrochemical stability with only a slight volume change (3–4%) even in a high current density (Wang et al., 2012). However, TiO_2_ is restricted by a low theoretical capacity (178 mAh/g), so TiO_2_ is often used as a supporting backbone or a protective layer for unstable active materials like SnO_2_ (Liu H. et al., 2015). Tian et al. have proposed a well-designed nanostructure where SnO_2_ particles are encapsulated in TiO_2_ hollow nanowires (Tian et al., 2014). The composite employs SnO_2_ embedded carbon nanowires as a template after being coated with TiO_2_ and calcinated in air. Void spaces between SnO_2_ particles and TiO_2_ shells have been demonstrated through TEM analysis. The voids offer space to accommodate volume changes of SnO_2_ nanoparticles during the charge/discharge process. With this unique yolk-shell structure and the role of TiO_2_ in the composite, the final SnO_2_@TiO_2_ composite exhibits a great cycle stability (445 mAh/g at a current density of 800 mA/g after 500 cycles). A summary of anode materials, synthetic methods, and electrochemical performances upon some SnO_2_-based anodes are pointed out in Table 2.

**Table 2:** Anode materials, synthetic methods and electrochemical performance of SnO_2_-based composites in LIBs.

**Anode materials**	**Synthetic method**	**ICE (%)**	**Cyclability (mAh/g)**	**Rate performance (mAh/g)**	**References**
Corn-like SnO_2_ nanocrystals/polydopamine	Combination of atom transfer radical polymerization, hydrothermal method and thermal treatment	61.3	1,494 (300 cycles at 160 mA/g)	835 (at 1A/g) 667 (at 2A/g)	Jiang B. et al., 2017
SnO_2_@TiO_2_	Hydrothermal synthesis and heat treatment	46.8	445 (500 cycles at 800 mA/g)	222 (at 1.6 A/g) 204 (at 2.0 A/g)	Tian et al., 2014
sSnO_2_@N-doped graphene	Hydrothermal treatment and thermal reduction	61.3	1,346 (500 cycles at 100 mA/g from)	631 (at 10 A/g)	Zhou et al., 2013b
SnO_2_ quantum dots@graphene oxide	Hydrothermal synthesis	about 53	112 (100 cycles at 100 mA/g)	417 (2,000 cycles at 2 A/g)	Zhao et al., 2016
F-doped SnO_2_@reduced graphene oxide (rGO)	Hydrothermal synthesis	60.5	1,037 (150 cycles at 100 mA/g)	860 (at 1 A/g) 770 (at 2 A/g)	Cui, 2017
Microwave-assisted SnO_2_@polypyrrole nanotube	Soft-template polymerization and microwave-assisted solvothermal synthesis	58.1	790 (200 cycles at 200 mA/g)	860 (at 1 A/g) 770 (at 2 A/g)	Du et al., 2016
SnO_2_@N-doped carbon fiber	Electrospinning and heat treatment	69.2	754 (300 cycles at 1,000 mA/g)	527 (at 1.6 A/g) 405 (at 3.2 A/g)	Xia et al., 2016

*ICE, Initial coulombic efficiency*.

### SnS_2_-Based Composites

Momma et al. and Brousse et al. have revealed that tin sulfides could also be used as novel anode materials in LIBs (Brousse et al., 1998; Momma et al., 2001). SnS_2_ materials possess superior physicochemical properties with a theoretical specific capacity of 645 mAh/g and a unique layered hexagonal CdI_2_-type crystal structure that is composed of tin cations sandwiched between two layers of close-packed sulfur anions in octahedral coordination, in which adjacent sulfur layers are linked with weak Van der Waals interactions and the interlayer intervals are about 0.59 nm (Morales et al., 1992; Lefebvre et al., 1997; Song et al., 2013; Deng et al., 2014; Li R. et al., 2019). Layer voids in this unique configuration are beneficial for the Li^+^ insertion process and can partially accommodate the volume change (Chen et al., 2017). However, integral volume changes and poor electronic conductivity of SnS_2_ are inevitable, which needs to be improved and one set of adopted electrochemical reactions have been put forward, which are the following (Momma et al., 2001; Kim et al., 2007):

(5) $$\begin{array}{l} \text{Sn}{\text{S}}_{2}+4\text{Li}+4{\text{e}}^{-}\to \text{Sn}+2\text{L}{\text{i}}_{2}\text{S} \end{array}$$

(6) $$\begin{array}{l} \text{Sn}+x\text{L}{\text{i}}^{+}+x\text{e}↔\text{Li}x\text{Sn}\left(0\leq x\leq 4.4\right) \end{array}$$

It can be obviously observed from the above equations that the reaction mechanism of SnS_2_ with lithium is very similar to the lithiation and delithiation of SnO_2_. In the first discharge cycle, metallic tin and amorphous Li_2_S are formed during the irreversible conversion of SnS_2_, where active Sn can be coated with the inactive Li_2_S, mitigating the volume changes of the electrode to some extent (Kim et al., 2009). With further charge and discharge processes, alloying/dealloying reactions of tin with lithium ions are reversible, but the capacity reduces rapidly due to the irreversible conversion and severe pulverization of SnS_2_ electrodes. Analogously, morphology design and the introduction of a conductive phase that accommodates volume changes, like amorphous carbon and graphene, can largely alleviate the volume changes of SnS_2_ in charge and discharge processes (Zhuo et al., 2012).

Since the microstructure of layered SnS_2_ materials has some resemblance to 2D graphene, the combination of them is more compatible than other dissimilar materials like SnO_2_, Sn, and Si (Bin et al., 2019). Few-layer SnS_2_/graphene hybrid materials synthesized using L-cysteine as a ligand in the solution-phase method have been reported by Chang et al. which can deliver a reversible specific capacity of 920 mAh/g at a current density of 100 mA/g (Chang et al., 2012). Additionally, graphene can be functionalized by doping with nitrogen, fluorine, or sulfur elements, and the doped graphene generates more defects and active sites which significantly enhances the electrochemical activity and conductivity (Guo et al., 2011). Zheng et al. have reported a large-scale and facile synthetic route for SnS_2_ nanoparticles coated with S-doped graphene (SnS_2_/S-rGO). The electrochemical stability of SnS_2_/S-rGO particles is much better than that of the undoped SnS_2_/rGO, in which the SnS_2_/S-rGO can possess a discharge specific capacity of 947 mAh/g whereas the SnS_2_/rGO is about 700 mAh/g after 200 cycles at 1A/g (Zheng et al., 2017). This result can be mainly ascribed to the stronger interaction of S-doped graphene with SnS_2_ particles.

**Figure 3:**
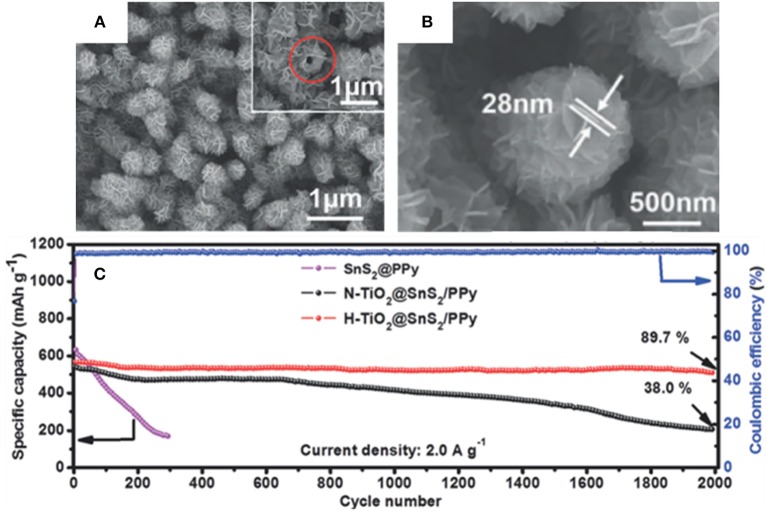
SEM images of H-TiO_2_@SnS_2_ **(A)** and H-TiO_2_@SnS_2_@PPy **(B)**, cycling performance **(C)** of SnS_2_@PPy, H-TiO_2_@SnS_2_@PPy, and N-TiO_2_@SnS_2_@PPy at 2.0 A/g. Reproduced from Wu et al. (2019) with permission from Copyright (2019) WILEY-VCH.

Wu et al. have presented a well-designed stable H-TiO_2_@SnS_2_@PPy composite by growing SnS_2_ sheets on hydrogen treated TiO_2_ (H-TiO_2_) nanowires and coating with carbonized polypyrrole (PPy), in which H-TiO_2_ provides some advantages over untreated TiO_2_. The key reason is that H-TiO_2_ structurally possesses more defects than the untreated TiO_2_, which provides increased conductivity and stronger chemical interactions with SnS_2_ (Ti-S) (Wu et al., 2019). Furthermore, the outermost carbonized PPy layer can accommodate the volume change to some degree as well as boosting the electronic conductivity. With the synergistic effects of the mentioned materials, the final H-TiO_2_@SnS_2_@PPy composite can deliver an outstanding electrochemical stability with a high discharge specific capacity of 508.7 mAh/g at 2.0 A/g after 2,000 cycles (Figure 3). A summary of anode materials, synthetic methods, and electrochemical performance of SnS_2_-based composites in LIBs have been displayed in Table 3.

**Table 3:** Anode materials, synthetic methods and electrochemical performance of SnS_2_-based composites in LIBs.

**Anode materials**	**Synthetic method**	**ICE (%)**	**Cyclability (mAh/g)**	**Rate performance (mAh/g)**	**References**
H-TiO_2_@SnS_2_@PPy	Combination of hydrolysis, hydrothermal route, thermal treatment and polymerization	71.2	508.7 (2,000 cycles at 2 A/g)	356.3 (at 10 A/g)	Wu et al., 2019
Few-layer SnS_2_/graphene	Hydrothermal method	42.4	920 (50 cycles at 100 mA/g)	520 (at 1 A/g)	Chang et al., 2012
SnS_2_/Sulfur doped graphene	Wet chemistry method	72	947 (200 cycles at 1 A/g)	550 (at 5 A/g)	Zheng et al., 2017
Porous vanadium nitride (VN)@SnS_2_	Hydrothermal method	77	819 (100 cycles at 650 mA/g)	349 (at 13 A/g)	Balogun et al., 2015
MoS_2_/SnS_2_-graphene oxide (GO)	One-pot hydrothermal synthesis	84.2	1,244 (190 cycles at 150 mA/g)	456 (at 3.8 A/g)	Jiang Y. et al., 2017
SnS_2_@PANI nanoplates	Hydrothermal and polymerization process	69.4	730.8 (80 cycles at 100 mA/g)	559.2 (at 2 A/g) 356.1 (at 5 A/g)	Wang G. et al., 2015
SnS_2_/graphene/ SnS_2_	Hydrothermal synthesis	81	1,357 (200 cycles at 100 mA/g)	844 (at 10 A/g)	Jiang et al., 2019

*ICE, Initial coulombic efficiency*.

## Tin and Tin Compounds in SIBs

The revival of sodium-ion batteries (SIBs) owes mainly to the low cost and abundance of sodium on earth. Although the intercalation mechanism of sodium and lithium are similar when used as electrodes in secondary alkali metal batteries, the larger radius size of Na^+^ (1.09 Å) compared to Li^+^ (0.74 Å) makes it challenging to find a suitable Na^+^ host with both excellent cycle stability and a relatively high capacity (Luo W. et al., 2016; Wu L. et al., 2018). Graphite is the most used anode in commercial LIBs but cannot insert Na^+^ effectively, which is due to the mismatching of graphite's interlayer interval (0.334 nm) with the larger radius of Na^+^ (Chevrier and Ceder, 2011). Moreover, Si is a very promising anode material for LIBs as it has a theoretical discharge specific capacity of 3,579 mAh/g, and some Si-based materials have been commercialized, but it cannot react with Na^+^ in the same manner as LIBs. This is because Na-induced lattice disturbance are remarkable in Si materials as they become endowed with small interstitial space and high stiffness (Chou et al., 2015; Fang et al., 2019). Interestingly, Sn, SnO_2_, and SnS_2_ can be applied in SIBs with relatively high capacity, low cost, and proper low charge/discharge potentials *vs*. Na/Na^+^, due to the minor Na-induced lattice disturbance in Sn-based materials (Guo et al., 2011; Zhu et al., 2013; Li et al., 2015). However, these active materials still go through huge volume changes, and the volume change is even severer in SIBs, which leads to serious pulverization of these brittle active materials ending up with rapid capacity decay and poor cycle stability (Ellis et al., 2012). The coping strategies of Sn, SnO_2_, and SnS_2_ in SIBs are analogous to ones in LIBs, which are the nanostructure design of these active materials and the process of simultaneously introducing a second phase that buffers the volume change (Nayak et al., 2018). Major improvements for Sn, SnO_2_, and SnS_2_ in SIBs have been separately detailed in the following sections and the anode materials, synthetic methods and electrochemical performance of Sn, SnO_2_, SnS_2_-based anode composites in SIBs are summarized in Table 4.

**Table 4:** Anode materials, synthetic methods and electrochemical performance of Sn, SnO_2_, and SnS_2_-based composite anodes in SIBs.

**Anode materials**	**Synthetic method**	**ICE (%)**	**Cyclability (mAh/g)**	**Rate performance (mAh/g)**	**References**
Sn_0.9_Cu_0.1_	Surfactant-assistant wet chemistry	—	420 (100 cycles at 169 mA/g)	126 (at 1.694 A/g)	Lin et al., 2013
Yolk-shell Sn_4_P_3_@C	Hydrothermal treatment and thermal reduction	43.8	515 (50 cycles at 100 mA/g)	421 (at 3 A/g)	Liu J. et al., 2015
SnSb/C composite	Mechanical milling	75.1	435 (50 cycles at 100 mA/g)	274 (at 1 A/g)	Xiao et al., 2012
Porous Ni_3_Sn_2_ microcages	Solvothermal reduction and crystallization	35.5	270 (300 cycles at 1A/g)	351 (at 5 A/g) 276 (at 10 A/g)	Liu J. et al., 2014
C@SnS/SnO_2_@graphene	Hydrothermal synthesis and sulfidation	74.6	713 (70 cycles at 30 mA/g)	550 (at 810 mA/g) 430 (at 2430 mA/g)	Zheng et al., 2016
MoS_2_@SnO_2_@C	Hydrothermal method and thermal treatment	67.99	396 (150 cycles at 50 mA/g)	168 (at 2 A/g)	Chen et al., 2018
SnO_2_@graphene	Hydrothermal synthesis	About 30.9	638 (100 cycles at 20 mA/g)	263 (320 mA/g) 143 (640 mA/g)	Su et al., 2013
Porous SnO_2_/Cu foil	Cold rolling method and anodization	73	326 (200 cycles at 200 mA/g)	232 (at 2 A/g) 150 (at 5 A/g)	Bian et al., 2016
Exfoliated SnS_2_/graphene	Sol-gel method and hydrothermal treatment	69	618.9 (100 cycles at 200 mA/g)	326 (at 4 A/g)	Liu Y. et al., 2014
SnS_2_/C nanospheres	Solid-state fabrication	about 54.5	600 (100 cycles at 50 mA/g)	360 (at 1 A/g)	Wang J. et al., 2015
SnS_2_/graphene/SnS_2_	Hydrothermal synthesis	66.8	1133 (100 cycles at 100 mA/g)	765 (at 10 A/g)	Jiang et al., 2019
TiO_2_@SnS_2_@Nitrogen-doped carbon	Combination of chemical synthesis in organic solution, hydrothermal synthesis and ALD	64.2	293 (600 cycles at 1 A/g)	152 (at 10 A/g)	Ren et al., 2018
Graphene/Sn@carbonaceous foam	Hydrothermal method and thermal reduction	About 55.1	434.2 (100 cycles at 100 mA/g)	166 (at 1.6 A/g) 3.2 (at 3.2 A/g)	Luo B. et al., 2016
MoS_2_/SnS_2_-graphene oxide (GO)	One-pot hydrothermal synthesis	76.5	655 (100 cycles at 150 mA/g)	550 (at 1.5 A/g) 340 (at 6.0 A/g)	Jiang Y. et al., 2017

*ICE, Initial coulombic efficiency*.

### Sn-Based Composites

The theoretical capacity of Sn as anode materials in SIBs (Na_15_Sn_4_) is about 847 mAh/g, but volume changes of Sn electrodes during charge-discharge processes are as high as 525 %, which is much higher than Sn in LIBs (Qian et al., 2014). As reported by Qian et al., the capacity of pure Sn electrodes in SIBs falls to zero in only five cycles, which can be explained by the pulverization of active materials during Na^+^ insertion/extraction processes (Ellis et al., 2013). Sn-based intermetallic alloy anodes have been demonstrated to be a reasonable solution to address the short cycle life of Sn (Li J. et al., 2019). A Sn-Cu alloy is a stable active/inactive alloy with a relatively high capacity in LIBs where the addition of Cu significantly increases the stability of the alloy. As mentioned in the LIBs section, the Cu_6_Sn_5_ alloy is more stable than other Sn-Cu intermetallics, but the application of Cu_6_Sn_5_ in SIBs is hampered by the short diffusion depth owing to the larger size of Na^+^. Regarding this, Lin et al. have reported using a Sn_0.9_Cu_0.1_ alloy in SIBs (Lin et al., 2013). In spite of a low initial discharge specific capacity of 250 mAh/g, the capacity gradually increased to 440 mAh/g in 20 cycles without capacity loss after 100 cycles.

**Figure 4:**
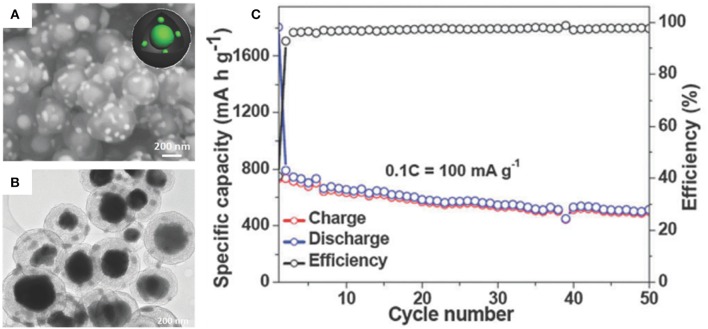
SEM **(A)** and TEM images **(B)** of yolk-shell Sn_4_P_3_@C. Cycling performance **(C)** of yolk-shell Sn_4_P_3_@C at 100 mA/g. Reproduced from Liu J. et al. (2015) with permission from Copyright (2015) Royal Society of Chemistry.

Sn-P intermetal is an emerging SIB anode material with balanced properties (Luo W. et al., 2016). Although the theoretical specific capacity of Sn_4_P_3_ (1,132 mAh/g) is significantly inferior to the pure P (2,560 mAh/g), electronic conductivity and theoretical volumetric capacity are much better than pure P in SIBs (Kim et al., 2014; Lan et al., 2017). Liu et al. have synthesized uniform yolk-shell Sn_4_P_3_@C nanoparticles for SIBs where Sn_4_P_3_ nanoparticles are encapsulated in hollow carbon spheres rendering some void space for the volume change of Sn_4_P_3_ whilst maintaining an intact microstructure (Liu J. et al., 2015). The carbon shell helps to form a stable SEI layer and strengthen the overall electronic conductivity of the composite. An initial discharge specific capacity of 790 mAh/g for yolk-shell Sn_4_P_3_@C nanospheres was determined and retained a high reversible specific capacity of 515 mAh/g after 50 cycles at 100 mA/g (Figure 4).

### SnO_2_-Based Composites

Sodiation/desodiation reactions of the SnO_2_ electrode are very similar to the lithiation/delithiation process, which include the conversion of SnO_2_ and reversible alloying/dealloying reactions contributing to the total theoretical specific capacity of 1,378 mAh/g (Su et al., 2013). SnO_2_ is one of the most extensively investigated anode materials in LIBs and nowadays, some of the SnO_2_-based composites have reached the theoretical capacity of SnO_2_ with an excellent cycle life. Herein, successful strategies in LIBs to address volume changes are advised for employment in SIBs as well (Chen and Lou, 2013).

Huang et al. have reported a facile *in situ* synthesis of 3D porous carbon encapsulated SnO_2_ nanoparticles (SnO_2_-PC) that exhibits a great cycle stability with a discharge specific capacity of 208.1 mAh/g at 100 mA/g after 250 cycles and SnO_2_-PC with a SnO_2_ weight percentage of 74.47 % demonstrated an extraordinary rate capability with a discharge specific capacity of 100 mAh/g at 1,600 mA/g after 1,000 cycles (Huang et al., 2016). The greatly improved electrochemical performance of the as-prepared SnO_2_-PC composite owes to the porous carbon matrix that can alleviate volume changes of SnO_2_ in the sodiation/desodiation process and improve the electronic conductivity of the composite.

**Figure 5:**
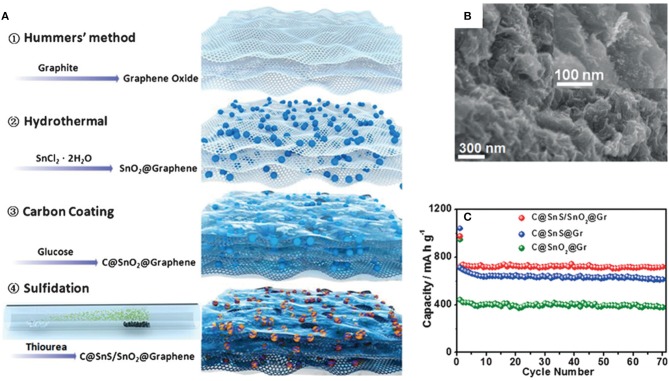
Schematic illustration **(A)** of the synthetic procedure of C@SnS/SnO_2_@graphene. SEM image **(B)** of C@SnS/SnO_2_@graphene. Cycling performance **(C)** of C@SnS/SnO_2_@graphene, C@SnS@graphene and C@SnO_2_@graphene at 30 mA/g. Reproduced from Zheng et al. (2016) with permission from Copyright (2016) WILEY-VCH.

Heterostructure has the advantage of high-speed electron transfer because of the interface effect. The heterojunction of nanocrystals with different band-gaps has been proven to enhance surface reaction kinetics and to provide increased charge transport. Zheng et al. have employed SnS in a C@SnO_2_@graphene composite in SIBs. The C@SnS/SnO_2_@graphene composite exhibits a high rate capability and long cycle life with a high capacity, which can be ascribed to the heterostructure of SnS/SnO_2_ which further improves the electronic conductivity and diffusion of Na^+^ in the electrode (Zheng et al., 2016). C@SnS/SnO_2_@graphene achieves a reversible discharge specific capacity of 713 mAh/g at 30 mA/g after 70 cycles, which is higher than C@SnS@graphene (around 600 mAh/g) and C@SnO_2_@graphene (around 400 mAh/g). By increasing the current density to 810 and 2,430 mA/g, the discharge specific capacity can be retained at 520 and 430 mAh/g, respectively (Figure 5).

### SnS_2_-Based Composites in SIBs

As mentioned, SnS_2_ has a special layered structure where tin cations are sandwiched between two layers of sulfur anions. The spacing between two adjoining two layers (d_002_ = 5.90 Å) is larger than the radius of Na^+^ (d_002_ = 1.09 Å), which allows the intercalation and diffusion of Na^+^ throughout the electrode effectively (Zheng et al., 2016). However, a pure SnS_2_ electrode contends with poor conductivity and severe pulverization. It has been demonstrated from previous studies that combining SnS_2_ with conductive materials will notably strengthen the electrochemical performance (Ren et al., 2017; Wu Y. et al., 2018). The unique 2D layer structure of SnS_2_ means it is highly compatible with graphene and can provide an increase in electronic conductivity. In 2014, Liu et al. discovered that exfoliated SnS_2_ restacked on graphene showed a remarkable electrochemical performance with a discharge specific capacity of 650 mAh/g at 200 mA/g after 100 cycles (Liu Y. et al., 2014). The excellent performance can be ascribed to the ultrasmall exfoliated-SnS_2_ layers being utilized fully when used as the electrode.

**Figure 6:**
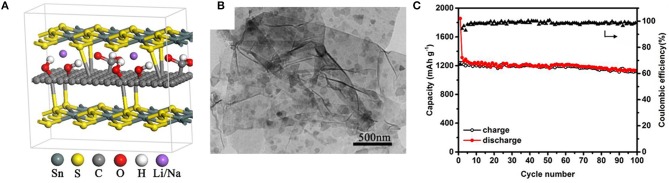
Molecular model **(A)** of sandwich like SnS_2_/graphene/SnS_2_; TEM image **(B)** of SnS_2_/graphene/SnS_2_; Cycling performance **(C)** of SnS_2_/graphene/SnS_2_ at 100 mA/g. Reproduced from Jiang et al. (2019) with permission from Copyright (2019) American Chemical Society.

Jiang et al. have reported a sandwich-like SnS_2_/graphene/SnS_2_ composite with expanded interlayers produced by a one-step hydrothermal synthesis, where both sides of the reduced graphene oxide sheets is covalently decorated with ultrathin SnS_2_ nanosheets (Jiang et al., 2019). The enlarged interlayer distance of SnS_2_ is about 8.03 Å, which assists the insertion/extraction of Li^+^/Na^+^ with rapid transport kinetics. As a result, SnS_2_/graphene/SnS_2_ composites have excellent electrochemical properties both in LIBs (see also in the LIBs section) and SIBs. To be specific for SIBs, the reversible discharge specific capacities of 1,295 mAh/g and 765 mAh/g are delivered at a current density of 0.1 and 10 A/g, respectively (Figure 6). Additionally, according to the structural characterizations of SnS_2_/graphene/SnS_2_ electrodes after 200 cycles, morphology changes and significant particle agglomeration cannot be clearly detected. Some reasons for the superiority of the SnS_2_/graphene/SnS_2_ composite are that the graphene sheet is sandwiched between SnS_2_ layers with enhanced conductivity and it has a strong structural integrity.

## Summary and Outlook

Sn, SnO_2_, and SnS_2_ have been extensively studied as substitutes for graphite in LIBs and for potential application in SIBs. Either in LIBs or SIBs, the ultimate problem that needs to be addressed is the huge volume change of Sn with Li^+^ or Na^+^ during the alloying/dealloying processes. This problem has been largely addressed by introducing one or more metals and/or compounds into the system and at least one additive which can act as an inactive buffering matrix. Also, the use of reasonable nanostructure design can tactfully mitigate the volume change and facilitate the diffusion of Li^+^ (Na^+^) and electrons. Due to these efforts, some of these tin-based anode materials have reached their maximum theoretical capacity. So far, the real practical uses of tin-based anodes is still very scarce in both LIBs and SIBs, which is mainly due to the tedious synthetic procedures, high costs and low yields. Recently, much work has focused on large-scale synthetic methods. We believe that a cost-effective and facile fabrication process which takes morphology into consideration can promote the application of tin-based anodes in commercial LIBs and large-scale energy storage equipment in SIBs.

## Author Contributions

HM and WX contributed conception and design of the study. CM, RL, and LY organized the database. HM wrote the first draft of the manuscript. WX revised the whole manuscript.

### Conflict of Interest

The authors declare that the research was conducted in the absence of any commercial or financial relationships that could be construed as a potential conflict of interest.
